# Innovating for Health: Measuring the Path of Global Innovation in Healthcare Systems

**DOI:** 10.3390/healthcare13233167

**Published:** 2025-12-03

**Authors:** Cristina Criveanu, Nicoleta Mihaela Doran, Veronica Gheorghiță, Oana Stăiculescu

**Affiliations:** 1 Department of Rheumatology, Faculty of Medicine, University of Medicine and Pharmacy of Craiova, 200349 Craiova, Romania; cristina.criveanu@umfcv.ro; 2 Department of Finance, Banking and Economic Analysis, Faculty of Economics and Business Administration, University of Craiova, 200585 Craiova, Romania; 3 Department of Sociology, Philosophy, and Social Work, Faculty of Social Sciences, University of Craiova, 200585 Craiova, Romania; veronica.gheorghita@edu.ucv.ro; 4 Department of Public Law and Administrative Sciences, Faculty of Law, University of Craiova, 200585 Craiova, Romania; oana.staiculescu@edu.ucv.ro

**Keywords:** innovation capacity, healthcare systems, quantile regression, European Union, healthy life expectancy

## Abstract

**Background/Objectives:** Innovation capacity has become a strategic pillar for strengthening healthcare systems in the European Union, yet its effects vary considerably across countries with different levels of institutional development and technological readiness. This study examines how national innovation capacity, measured through the Global Innovation Index, influences health expenditure, healthy life expectancy, and childhood obesity across the EU-27. **Methods:** Using an unbalanced panel dataset for 2011–2024, we applied panel quantile regression to capture heterogeneous effects across the conditional distribution of health outcomes. Four dependent variables were analyzed: government expenditure on health, provider-level healthcare spending, healthy life expectancy at birth, and childhood obesity prevalence. GDP growth and population were included as controls. Diagnostic tests confirmed cross-sectional dependence and heteroskedasticity, supporting the choice of distributionally robust estimators. **Results:** Higher innovation capacity was positively and significantly associated with government health expenditure and provider-level spending across all quantiles (*p* < 0.001), with the strongest effects in lower-performing systems. For healthy life expectancy, innovation exhibited declining coefficients across quantiles, indicating diminishing marginal returns in more advanced systems. No stable association was observed for childhood obesity, which remained largely unaffected by national innovation capacity. **Conclusions:** Innovation contributes to structural improvements in health financing and population health, particularly in countries with lower baseline performance. In high-performing systems, its role shifts toward incremental efficiency gains. The absence of effects on childhood obesity highlights the dominance of socio-behavioral determinants. Findings are associative and call for future causal and sector-specific research.

## 1. Introduction

In recent years, health systems worldwide have undergone profound transformation driven by rapid technological progress, demographic change, and increasing demand for equitable and efficient care. Across the European Union (EU), aging populations, rising chronic disease burdens, and fiscal pressures continue to challenge the sustainability of healthcare provision [1,2]. The COVID-19 pandemic further demonstrated the critical role of innovation—particularly digital tools, coordinated governance, and research capacity—in strengthening system resilience [3]. As highlighted by the European Commission, sustainable healthcare depends not only on financial inputs but also on a country’s ability to generate, diffuse, and apply innovation [4].

Innovation has therefore become a central pillar of EU strategic initiatives, including the European Innovation Agenda and EU4Health, which link innovation, digital transformation, and resilience objectives [5,6]. Similarly, the World Health Organization frames innovation as essential to achieving Universal Health Coverage and advancing the Sustainable Development Goals, notably SDG 3 and SDG 9 [7].

The Global Innovation Index (GII), developed by WIPO, provides a comprehensive framework assessing national innovation capacity across institutions, human capital, infrastructure, knowledge creation, and technology diffusion [8]. In this study, the term global innovation refers strictly to the conceptual and methodological scope of the GII—its multidimensional measurement framework—not the geographical coverage of the empirical dataset [9]. Although the GII allows comparison across a global sample, the current research employs it as a national-level proxy for innovation capacity within the EU context, fully consistent with its methodological design.

Despite growing policy emphasis on innovation in healthcare, empirical evidence on the macro-level link between national innovation capacity and health outcomes remains limited. Existing studies predominantly examine micro-level adoption—such as hospital technologies, pharmaceutical innovation, or telemedicine implementation [10,11,12]—without capturing systemic effects on expenditure, population health, or preventive outcomes. Moreover, innovation is rarely integrated into econometric analyses alongside public health indicators, leaving unexplored the broader relationship between national competitiveness, demographic pressures, and healthcare efficiency [13].

Few cross-national studies incorporate innovation metrics such as the GII as explanatory variables in models of health expenditure or population health [14,15], and none to our knowledge systematically quantify these relationships across all 27 EU Member States using harmonized datasets.

The empirical analysis focuses exclusively on the 27 European Union Member States due to their high degree of institutional comparability, harmonized statistical reporting, and shared strategic policy frameworks in both innovation and healthcare. The EU represents a distinct policy ecosystem in which data collection follows standardized methodologies—such as the System of Health Accounts (SHA 2011), Eurostat classifications, and WHO-Europe monitoring systems—allowing for methodologically consistent cross-country comparisons that are not feasible in a global sample where governance, data availability, and institutional capacities differ substantially.

Moreover, the EU has adopted coordinated mechanisms linking innovation to public health objectives, including EU4Health, Horizon Europe, and the European Innovation Agenda, which position innovation as a systemic driver of resilience, interoperability, and sustainable health outcomes. Studying the EU as an integrated unit therefore enables a focused examination of how innovation translates into healthcare performance in a region characterized by common regulatory principles and synchronized strategic priorities, while future research may expand the scope to additional regions for comparative validation.

This study examines the extent to which national innovation capacity influences healthcare system performance in the EU from 2011 to 2024. We hypothesize that higher innovation capacity—as measured by the GII—acts as a systemic driver through three channels: (1) increased and better-targeted public health expenditure; (2) improved provider-level resource allocation and system efficiency; and (3) enhanced population health outcomes. The first channel reflects how innovation ecosystems strengthen strategic planning, evidence generation, and technological procurement, leading to higher and better-targeted public health expenditure. The second channel captures how organizational and technological innovation enhances coordination across healthcare providers, digital infrastructure, and service delivery models. The third channel relates to long-term improvements in life expectancy and overall well-being driven by advances in biomedical research, diagnostics, and treatment. By contrast, childhood obesity is included as a behavioral indicator whose determinants are expected to extend beyond technological innovation.

## 2. Literature Review

Healthcare innovation represents a multidimensional construct that encompasses technological, organizational, and social processes designed to enhance healthcare efficiency, quality, and accessibility. The European research agenda increasingly views innovation as a systemic process involving collaboration between policy-makers, practitioners, and industry actors [16]. Andrews et al. [16] emphasized that while the European Union has long promoted pre-commercial procurement (PCP) and public procurement of innovation (PPI) frameworks, their practical implementation in healthcare remains limited, especially in digital transformation contexts. Kovács [17] observed that in Central and Eastern European clusters, innovation depends less on state subsidies and more on intra-cluster learning, absorptive capacity, and trust-based collaboration, suggesting that innovation success derives from strategic integration rather than regulatory intervention.

Szentpeteri et al. [18] proposed a multidisciplinary framework integrating medical, engineering, and management expertise to increase the efficiency of healthcare innovations, emphasizing that cross-functional collaboration enhances the translation of ideas into market-ready solutions. Likewise, Wirth et al. [19] explored social innovation through the lens of “change agency,” revealing how collective leadership and stakeholder co-creation foster systemic transformation. Overall, these studies converge on the notion that healthcare innovation extends beyond technological advances—it involves networked knowledge creation, institutional learning, and systemic coordination.

Digital transformation has become the cornerstone of healthcare modernization, redefining how medical services are designed, delivered, and evaluated. Schiavone and Omrani [20] argued that responsible digital transformation requires balancing open innovation with ethical and regulatory constraints, a challenge particularly visible in data-driven medicine. Santarsiero et al. [21] highlighted the pivotal role of innovation labs as learning environments that accelerate digital transformation by fostering experimentation and user-driven design within hospitals.

Similarly, Krychowski and Du Montcel [22] demonstrated that integrating open innovation and additive manufacturing within hospitals fosters new forms of medical device production, shortening development cycles and empowering clinicians as co-innovators. Akinwale and AboAlsamh [23] provided quantitative evidence linking technological innovation (AI, telehealth, and EHR systems) with improved operational efficiency and healthcare performance. Denicolai and Previtali [24] further found that a well-defined innovation strategy mediates the relationship between digital transformation and organizational effectiveness, emphasizing governance and alignment as success factors. Collectively, these studies establish that digital transformation depends as much on cultural readiness and leadership as on technological adoption.

Organizational culture profoundly shapes how healthcare institutions absorb and deploy innovation. Klassen et al. [25] developed a multidimensional construct for measuring innovation culture, identifying leadership commitment, knowledge sharing, and motivation as primary enablers. Moisoglou et al. [26] revealed that innovation-supportive environments mitigate “quiet quitting” and enhance creativity among nurses, demonstrating that managerial support directly influences individual-level innovative behavior.

Manfrini and Bäckström [27] explored employee-driven innovation (EDI) in Italy’s public healthcare sector, showing that empowering front-line staff increases service quality and organizational learning capacity. Nowak [28] highlighted the detrimental effects of resistance to change, which weakens absorptive capacity and reduces the organization’s ability to implement new ideas. Collectively, these findings underscore that fostering a supportive innovation climate—rooted in trust, autonomy, and leadership—constitutes a prerequisite for sustainable transformation in healthcare organizations.

The literature increasingly emphasizes innovation ecosystems and cross-sector collaboration as critical enablers of healthcare transformation. The Platform for Innovation of Procurement and Procurement of Innovation (PiPPi) project provides a prominent example of a European community of practice connecting hospitals, research agencies, and suppliers to address unmet healthcare needs [16]. Sultana and Turkina [29] demonstrated that intermediaries play a pivotal role in orchestrating sustainable innovation ecosystems by aligning stakeholders and promoting iterative learning toward the Sustainable Development Goals (SDGs).

Pikkarainen et al. [30] examined global collaboration mechanisms in healthcare innovation ecosystems, revealing that universities act as key orchestrators, facilitating funding access, regulatory alignment, and knowledge diffusion. Oliveira et al. [31] showed that applying Design Thinking in hospital settings accelerates organizational learning, fostering user-centered innovation outcomes. These studies collectively confirm that healthcare innovation thrives in open, networked ecosystems that enable resource sharing, experimentation, and adaptive governance.

Despite the growing emphasis on innovation, numerous barriers persist at institutional, regulatory, and behavioral levels. Madanaguli et al. [32] identified individual-level barriers among Swedish healthcare professionals—such as perceived uncertainty and lack of organizational support—that hinder technological adoption. Similarly, Kumari et al. [33] classified the main obstacles to open innovation in healthcare into performance, legal, and value-related barriers, noting that inadequate risk-sharing mechanisms frequently undermine innovation initiatives.

Manfrini and Duff [34] analyzed the emotional and ethical dimensions of innovation, showing that compassion and care practices can serve as non-technological drivers that enhance innovation acceptance. Rake et al. [35] argued that the COVID-19 pandemic catalyzed new models of collaboration in medical R&D, revealing the potential for adaptive innovation governance in times of crisis. These findings collectively demonstrate that innovation success is conditioned by institutional flexibility, professional motivation, and inter-organizational coordination.

Although research on healthcare innovation has expanded substantially, significant gaps remain. Stoumpos et al. [36] conducted a bibliometric analysis showing limited integration between knowledge management and digital innovation, advocating for more interdisciplinary studies to bridge theoretical and practical divides. Cano et al. [37] emphasized that many hospitals lack systematic tools to assess innovation maturity, proposing the “Innovation Map” as an effective model for evaluating awareness, engagement, and outcomes.

Gómez-Caballero [38] identified the need for clearer conceptualization of “innovation competence” within healthcare services, particularly concerning patient experience and co-creation. Future research should focus on quantifying the causal links between innovation inputs (training, R&D investment, digital adoption) and health system outcomes (accessibility, patient safety, and sustainability). Moreover, as digitalization accelerates, scholars must address ethical governance, data security, and inclusivity to ensure that technological advancement translates into equitable healthcare access across Europe.

The reviewed literature highlights that innovation influences healthcare systems through multiple systemic channels rather than isolated technological interventions. Existing studies emphasize the role of innovation in strengthening governance mechanisms, institutional coordination, digital infrastructure, procurement models, and clinical processes, yet findings remain fragmented and often context-specific.

**Figure 1:**
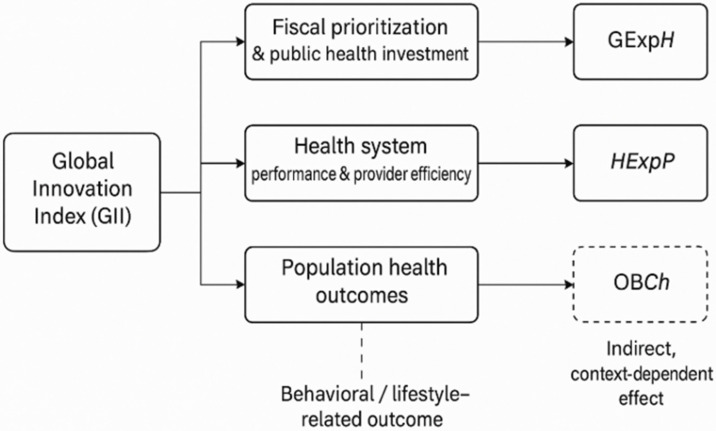
Conceptual framework linking national innovation capacity to healthcare performance in the EU.

To consolidate these insights and derive a coherent analytical structure, this study synthesizes theoretical contributions into a unified conceptual framework that maps how national innovation capacity—measured through the Global Innovation Index—translates into fiscal prioritization, system-level performance improvements, and population health outcomes, while exerting a more limited influence on behavioral health indicators such as childhood obesity. The framework below (Figure 1) integrates these causal pathways and informs the subsequent empirical model, enabling a structured examination of heterogeneous effects across EU Member States.

Building upon the conceptual framework presented in Figure 1 and grounded in the existing body of literature on innovation-driven health system transformation, the following hypotheses are formulated:

**H1.**  *Greater national innovation capacity is positively associated with government expenditure on domestic health, reflecting enhanced strategic prioritization, institutional planning, and evidence-based resource allocation.*

**H2.**  *Higher innovation capacity is positively associated with healthcare provider expenditure per capita, indicating system modernization, digital integration, and organizational adaptation enabled by innovation ecosystems.*

**H3.**  *National innovation capacity is positively associated with improvements in population-level health outcomes, as reflected in higher healthy life expectancy at birth, driven by advances in biomedical research, diagnostics, and clinical capability.*

**H4.**  *National innovation capacity exhibits no substantial direct effect on behavioral and lifestyle-dependent health outcomes, such as childhood obesity, due to the predominance of socioeconomic, cultural, and individual behavioral determinants.*

## 3. Materials and Methods

### 3.1. Data Selection

This study employs an unbalanced panel dataset including the 27 Member States of the European Union (EU-27) observed over the period 2011–2024. The temporal scope was chosen to encompass major structural transitions that shaped European healthcare systems, including the post-financial crisis recovery, the digitalization initiatives under the Horizon 2020 framework, and the post-pandemic reforms triggered by the COVID-19 crisis. This extended timeframe provides a consistent empirical basis to examine how national innovation capacity has influenced healthcare performance in a context of growing economic, demographic, and institutional diversity. All data were collected from internationally recognized and publicly accessible sources, as presented in Table 1.

The panel used in this study is unbalanced due to variations in reporting frequency across international databases. All indicators were included strictly based on reported annual values, with no interpolation or extrapolation applied. Missing observations were omitted rather than imputed to ensure data integrity and avoid introducing artificial trends. As a result, the number of observations varies across indicators, especially for variables such as Healthy Life Expectancy (HLE) and Obesity in Children (OBCh), which are not reported annually by all countries. Given the variation in reporting frequency across years for several indicators, each regression is estimated on the maximally available sample for the corresponding dependent variable. Consequently, the number of observations differs across models, and coefficient magnitudes should be interpreted within each indicator rather than compared directly across different indicators.

The empirical analysis focuses on four dependent variables that jointly capture both the financial commitment of governments to health and the population-level outcomes of healthcare systems. The first, General Government Expenditure on Domestic Health (GExpH), represents the percentage of total government spending allocated to health and reflects the fiscal prioritization of healthcare within national budgets. This indicator serves as a proxy for the public sector’s capacity and willingness to invest in citizens’ well-being. The second dependent variable, Healthcare Expenditure by Provider (HExpP), measures the distribution of current health expenditure across types of healthcare providers such as hospitals, outpatient centers, and preventive institutions. Together, GExpH and HExpP provide a multidimensional picture of the financial structure and efficiency orientation of EU health systems.

To capture the population-health dimension, the analysis includes Healthy Life Expectancy at Birth (HLE), expressed in years, which measures the average number of years that a newborn is expected to live in good health, taking into account both morbidity and mortality patterns. This indicator represents a widely used composite measure of healthcare quality and accessibility. A second outcome variable related to public health is OBCh, expressed as the percentage of the population in this age group classified as obese. This indicator acts as a proxy for preventive healthcare efficiency and health education effectiveness, offering an outcome-based measure of how innovation and policy translate into healthier lifestyles. Childhood obesity is included as a behavioral outcome intended to contrast the effects of national innovation capacity on systemic health performance indicators with outcomes driven primarily by individual behavior, socioeconomic factors, and cultural determinants. Unlike life expectancy or health expenditure, obesity rates reflect lifestyle and social conditions, and therefore provide a benchmark to assess whether innovation can indirectly affect preventive and behavioral health metrics rather than structural outcomes.

The study’s key independent variable is the GII, published annually by WIPO. The GII provides a composite score ranging from 0 to 100 and summarizes national innovation capacity across seven dimensions: institutions, human capital and research, infrastructure, market sophistication, business sophistication, knowledge and technology outputs, and creative outputs. The index offers an integrated view of both the inputs and outputs of innovation, thereby capturing countries’ ability not only to invest in research and technology but also to generate applicable knowledge, patents, and creative solutions. Data were collected for the entire period 2011–2024 from WIPO’s official reports. Because the Global Innovation Index uses a harmonized framework, it is fully comparable across countries and time, making it an appropriate proxy for assessing the national innovation environment in the European context.

To account for structural differences in macroeconomic performance and demographic scale, two control variables were included: GDP per Capita Growth (GDP) and Population (POP). The GDP growth rate, expressed as an annual percentage, measures the rate of expansion of national economies and reflects fiscal capacity to sustain health investments. Economic growth is a well-established determinant of healthcare spending and system resilience, influencing both government expenditure and private sector participation. The population variable, measured in millions of inhabitants, accounts for country size and demographic pressure, which directly affect both the demand for healthcare services and the scalability of innovation adoption. Together, these controls ensure that the estimated relationships between innovation and health outcomes are not confounded by differences in economic development or population structure.

### 3.2. Methodology

**Figure 2:**
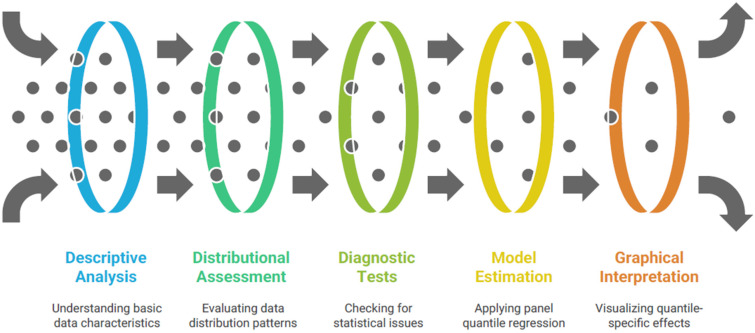
Methodological framework for analysis.

The empirical strategy employed in this study combines descriptive statistical analysis, diagnostic testing, and advanced panel econometric techniques to assess the relationship between innovation capacity and healthcare performance across the 27 EU Member States during 2011–2024. The analysis proceeds through five main stages as presented in Figure 2. This methodological framework ensures robustness, distributional flexibility, and an accurate depiction of heterogeneous relationships between innovation and healthcare outcomes.

The first stage involves a descriptive statistical analysis of all variables, including measures of central tendency (mean, median), dispersion (standard deviation, variance), and distributional shape (skewness and kurtosis). Descriptive statistics provide initial insights into the structure and variability of the data, enabling the identification of potential outliers and country-specific heterogeneities. The results are complemented by a kernel density estimation (KDE), which visualizes the empirical distribution of the main variables—particularly health expenditure and innovation scores—allowing the detection of non-normality and asymmetric patterns across the EU sample. The KDE approach is nonparametric and relies on the probability density function to describe how observations are distributed around the mean, serving as a pre-estimation diagnostic for subsequent quantile-based methods [46].

In the second stage, before model estimation, diagnostic tests are applied to assess the presence of cross-sectional dependence, heteroskedasticity, and serial correlation—common issues in macro-panel datasets where economic and health variables may be interlinked across countries.

Cross-sectional dependence is tested using the Pesaran (2004, 2015) CD and bias-adjusted LM tests, which evaluate whether residuals from different cross-sections (countries) are correlated beyond random variation [47,48]. A significant statistic indicates interdependence among EU Member States, justifying the use of robust covariance estimators that account for such correlations. The presence of heteroskedasticity is examined using the Breusch–Pagan (1979) Lagrange Multiplier (LM) test, which detects variance heterogeneity across units and time [49]. Serial correlation is checked using the Drukker (2003) test for panel data models, which ensures that model residuals are temporally independent [50]. These diagnostic procedures are essential to validate the statistical assumptions of panel quantile models and to avoid biased inference.

Once the diagnostic checks confirm the existence of cross-sectional dependence and heteroskedasticity—phenomena typically observed in macro-level EU data due to policy synchronization and structural diversity—the study proceeds to estimate Panel Quantile Regression (PQR) models. Traditional mean-based estimators such as Fixed Effects (FE) or Random Effects (RE) regressions provide limited insight into heterogeneous effects across the conditional distribution of the dependent variable. In contrast, quantile regression allows the estimation of relationships at different points (quantiles) of the conditional distribution, thereby capturing asymmetries and heterogeneity among countries with distinct healthcare profiles [51,52]. Originally introduced by Koenker and Bassett [51], quantile regression minimizes a weighted sum of absolute residuals rather than squared residuals, offering robustness to outliers and non-normal error distributions.

To justify the choice of panel quantile regression, we emphasize that countries within the European Union exhibit substantial heterogeneity in both innovation capacity and healthcare performance, leading to non-uniform responses across the conditional distribution of outcomes. Mean-based estimators such as OLS or FMOLS assume homogeneous effects and may obscure differences between countries positioned at lower versus higher performance levels. In contrast, panel quantile regression enables the estimation of heterogeneous marginal effects across quantiles, capturing how innovation influences health outcomes differently in systems characterized by underperformance compared to those with already developed institutional capacity and higher innovation absorption potential. We employ panel quantile regression because it captures heterogeneous effects across the distribution of health outcomes, avoids restrictive mean-based assumptions, and is robust to non-normality and heteroskedasticity, which are clearly present in our dataset.

The general form of the panel quantile regression model estimated in this study can be expressed as follows:

(*τ*∣*X*_*it*_) = (*τ*) + *X*_*it*_′*β*(*τ*) + *ε*_*it*_(*τ*) (1)

where *Q*_*Yit*_(*τ*∣*X*_*it*_) denotes the conditional *τ*-th quantile (*τ* = 0.10, …, 0.90) of the dependent variable (e.g., health expenditure or life expectancy) for country *i* at time *t*; *X*_*it*_ is the vector of explanatory variables including the GII, GDP growth, and population; *α*_*i*_(*τ*) represents country-specific fixed effects that capture unobserved heterogeneity; and *β*(*τ*) denotes the quantile-specific slope parameters. Estimation is conducted using the Koenker (2004) approach for longitudinal data, which extends the classical quantile regression framework to panel structures by incorporating fixed effects [52]. The coefficients (*τ*) are interpreted as the marginal effects of innovation on healthcare outcomes at different quantiles of the distribution.

Panel quantile regression offers several advantages over mean regression methods. First, it is robust to heteroskedasticity and non-normality of residuals [51,53]. Second, it captures distributional heterogeneity, revealing whether the effect of innovation differs between countries with low and high healthcare performance. Third, it allows for flexible inference across quantiles, providing a richer understanding of the complex and potentially nonlinear link between innovation and health system outcomes [54]. To ensure valid inference, standard errors are computed using the Buchinsky (1995) method, which provides an asymptotically consistent covariance matrix estimator for quantile regressions through bootstrap replications [55].

After model estimation, the Likelihood Ratio (LR) Heteroskedasticity Test and the Residual Cross-Section Dependence Test are repeated on the quantile regression residuals to validate the robustness of the estimates. The LR test examines whether variance differences across cross-sections remain significant, while the CD test checks if unobserved interdependencies persist in the residual structure [47,49]. Visual inspection is provided by plotting the estimated coefficients across quantiles, forming a quantile process graph that illustrates how the marginal effect of innovation varies across different levels of healthcare performance. This graphical representation allows the identification of nonlinearities and threshold effects, showing whether innovation exerts stronger impacts on countries with either low or high health expenditure shares.

The bidirectional relationship between innovation capacity and healthcare outcomes introduces potential endogeneity, as higher innovation may improve population health, while better health strengthens human capital and institutional capacity, reinforcing innovation adoption. To mitigate simultaneity concerns, the model includes country fixed effects, which control for unobserved time-invariant heterogeneity, and incorporates lagged values of the Global Innovation Index as robustness checks to reduce temporal feedback effects. While these measures limit endogeneity risks, we acknowledge that complete causal isolation is not feasible within the current data structure, and the results should be interpreted as associative rather than strictly causal.

The set of control variables was intentionally kept parsimonious to avoid multicollinearity and overfitting, given the limited availability of harmonized annual data for all EU Member States. GDP per capita growth captures the macroeconomic context influencing both innovation and public health spending, while population size reflects structural differences in system scale and resource allocation. We recognize that additional controls—such as education levels, income inequality, labor market composition, or welfare expenditures—may further improve model specification; however, these variables are inconsistently available across the full panel and would significantly reduce the number of usable observations. The present model should thus be viewed as an exploratory baseline, to be extended in future research once broader harmonized data become available.

All statistical analyses are conducted using EViews 12 (S&P Global, 2021), which includes dedicated tools for quantile regression estimation, panel data handling, and diagnostic testing [56]. The software enables the computation of robust standard errors, kernel density graphs, and quantile-specific confidence intervals. The methodological framework applied in this study follows established econometric literature, combining the foundational works of Koenker and Bassett [51], Koenker (2004, 2005) [52,53], and subsequent extensions for dependent data structures, alongside diagnostic testing procedures by Pesaran (2004, 2015) [47,48], Breusch and Pagan (1979) [49], Drukker (2003) [50], and Wooldridge (2010) [54].

## 4. Results

The descriptive statistics, presented in Table 2, reveal substantial heterogeneity across EU Member States in terms of healthcare resources, innovation capacity, and demographic scale.

Public expenditure on domestic health averages 13.5% of GDP, with relatively moderate dispersion (SD ≈ 3.03), indicating shared fiscal commitments to public healthcare; however, values range widely—from below 6% in lower-spending systems to nearly 21% in highly investment-oriented states—suggesting significant variability in budgetary prioritization.

Healthcare provider expenditure per capita exhibits far greater dispersion (SD ≈ 1674), with a six-fold ratio between minimum and maximum values, signaling structural inequality in service provision and resource allocation.

Healthy life expectancy (HLE) shows lower variability (mean ≈ 68.8 years; SD ≈ 2.1), implying convergence in population-level outcomes despite differences in spending. In contrast, childhood obesity rates vary notably (range 3.7–13.3%), reflecting lifestyle, cultural, and socio-economic determinants rather than system financing alone.

GII displays a balanced distribution (mean ≈ 48.9; SD ≈ 7.4), consistent with relatively aligned institutional frameworks across the EU. GDP per capita growth exhibits extreme values (from −11.39% to +23.44%), reflecting cyclical economic shocks—particularly during crises such as the COVID-19 pandemic—while population size demonstrates the highest skew (skewness 1.79) and variance due to structural disparities between small and large Member States.

The normality diagnostics indicate mixed distributional properties across variables. Jarque–Bera tests confirm non-normality for most indicators (*p* < 0.01), including HExpP, HLE, OBCh, GII, GDP growth, and population—supporting the use of econometric techniques robust to non-Gaussian distributions, such as quantile regression. GExpH is the only variable approximating normal distribution (*p* ≈ 0.35), reflecting its more uniform policy alignment across states. Skewness patterns further highlight structural dynamics: HLE is negatively skewed, suggesting clustering toward higher life expectancy levels, while population and GDP display right-skewed tails driven by outlier countries with large populations and atypical economic volatility. Kurtosis values above 3 for GDP and population indicate heavy-tailed distributions, reinforcing the need for distribution-sensitive models rather than mean-based estimators. Overall, the descriptive evidence supports the empirical strategy by demonstrating that health outcomes, expenditures, and innovation capacity vary meaningfully across member states and cannot be adequately summarized through central tendency measures alone.

**Figure 3:**
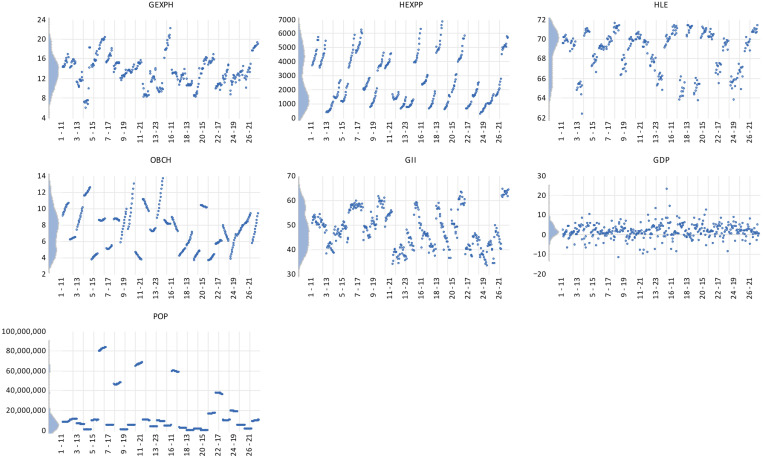
Kernel density graph.

The kernel density distributions presented in Figure 3 provide a visual overview of the empirical shape and dispersion of the variables used in the analysis, highlighting the degree of heterogeneity across EU Member States. The estimated densities for the healthcare indicators—GExpH, HExpP, and HLE—reveal distributions that are moderately concentrated around their respective means, suggesting relative stability and convergence of health financing and outcomes within the EU. The curve for general government expenditure on health displays a slight right skewness, implying that a smaller group of countries allocate substantially higher shares of public spending to healthcare. In contrast, the distribution of healthy life expectancy is nearly symmetrical, indicating that most Member States cluster around similar health outcome levels, with limited polarization.

The distribution of OBCh and GII presents more pronounced asymmetries. The right-tailed shape of the OBCh density function indicates that a few countries maintain significantly higher obesity prevalence rates compared to the EU average, reflecting persistent challenges in preventive healthcare and lifestyle management. Meanwhile, the GII kernel curve shows a smoother, left-skewed distribution, suggesting that a larger proportion of countries are positioned below the EU innovation frontier, while only a small group consistently achieves top innovation scores. This imbalance underlines the structural innovation divide between northern and western Europe—characterized by advanced research and digital infrastructures—and the southern and eastern regions, which continue to close the gap gradually.

For the control variables, GDP growth and population size (POP), the distributions demonstrate the expected asymmetry associated with macroeconomic heterogeneity. GDP growth density is tightly centered near zero, indicating limited volatility in real economic performance across the EU, whereas the population density is sharply right-skewed, reflecting the demographic dominance of a few large states such as Germany, France, and Italy. Overall, the kernel density analysis confirms that while health and innovation variables show moderate convergence, structural asymmetries persist across Member States—justifying the use of quantile-based estimation techniques to capture heterogeneous effects of innovation on healthcare performance along the conditional distribution.

The results reported in Table 3 reveal a significant degree of cross-sectional dependence across the 27 EU member states for all the analyzed variables. All three diagnostic tests, namely the Breusch–Pagan LM, Pesaran scaled LM, and Pesaran CD, reject the null hypothesis of cross-sectional independence at the 1% significance level (*p* < 0.01). This strong correlation among residuals implies that shocks, policy measures, or macroeconomic fluctuations in one EU country tend to spill over into others, reflecting the high degree of policy integration and shared institutional frameworks within the European healthcare and innovation systems.

These findings underscore that EU healthcare systems operate within an interconnected policy and economic environment, where the diffusion of medical technologies, funding mechanisms, and health innovation policies often transcends national boundaries. The particularly high dependence observed for HExpP and OBCh indicates that both financial and behavioral health dimensions are jointly influenced by supranational policies and regional dynamics. Consequently, the rejection of the null hypothesis confirms that panel quantile regression techniques accounting for cross-sectional dependence are methodologically justified, as they provide more robust and reliable estimates in the presence of such inter-country correlations.

Table 4 provides clear evidence of heteroskedasticity across cross-sections for all the dependent variables. The Likelihood Ratio (LR) test statistics are highly significant (*p* < 0.01), leading to the rejection of the null hypothesis of homoscedastic residuals. This finding indicates that the variance of the error terms is not constant across EU Member States, suggesting that the magnitude of fluctuations in healthcare-related indicators varies depending on national characteristics such as economic development, institutional efficiency, and policy priorities.

From an econometric perspective, the presence of heteroskedasticity confirms that a uniform-variance assumption would lead to inefficient and potentially biased estimates if traditional ordinary least squares (OLS) or fixed-effects methods were applied. Instead, the observed heterogeneity in variance justifies the adoption of robust estimation techniques such as panel quantile regression, which are inherently resistant to non-constant error variance and outlier sensitivity. In substantive terms, the heteroskedastic structure reflects the reality of the European healthcare landscape—where smaller economies or those undergoing systemic reform experience greater volatility in health financing and outcomes compared to larger, more stable systems. This reinforces the relevance of employing quantile-based approaches that can capture distributional asymmetries and heterogeneity in the innovation–healthcare nexus across the EU.

The results in Table 5 reveal significant and nuanced relationships between innovation performance and the four healthcare indicators analyzed across EU Member States, underscoring the multifaceted impact of technological and institutional innovation on health systems.

The first relationship, between the GII and GExpH is strongly positive and statistically significant (*p* < 0.01), indicating that higher innovation performance is associated with a greater fiscal commitment to public health. This suggests that innovation-oriented countries, typically those with strong institutional capacity and effective governance frameworks, prioritize health spending as part of their broader social investment strategies. In such economies, innovation enhances administrative efficiency and budgetary planning, enabling more targeted allocation of resources toward preventive care, medical research, and healthcare infrastructure. The positive link therefore reflects the strategic alignment between innovation policy and public health governance in the European Union, where technology-driven modernization supports the sustainability and inclusiveness of health systems. Moreover, this finding underscores the importance of innovation as a policy catalyst, fostering not only technological advancement but also long-term fiscal responsibility in the health sector.

The second relationship, between GII and HExpP, also exhibits a significant and positive coefficient (*p* < 0.01), although with a lower magnitude compared to GExpH. This association implies that innovation stimulates greater efficiency and structural diversification within healthcare delivery systems. Innovative countries tend to adopt advanced management technologies, digital patient record systems, and telemedicine solutions that transform how healthcare providers operate and deliver services. The diffusion of innovation enhances coordination across hospitals, outpatient centers, and primary care facilities, leading to improved service quality and reduced duplication of costs. Thus, the positive relationship reflects how technological diffusion and organizational innovation reshape the operational dynamics of healthcare provision, enabling EU Member States to transition toward more resilient and digitally integrated health systems. In practical terms, innovation serves as an enabler of modernization, allowing countries to move from volume-based to value-based healthcare delivery.

The third relationship, between GII and healthy life expectancy at birth (HLE), demonstrates a strong and statistically significant positive effect (*p* < 0.01), underscoring the long-term social benefits of innovation. Higher innovation capacity is linked to improved population health outcomes, as it enhances both the accessibility and quality of medical services. Innovation fosters the development and diffusion of advanced diagnostic tools, biotechnologies, and digital health applications that contribute to early disease detection and more effective treatment. Additionally, countries with higher GII scores typically invest more in health-related research and education, generating spillover effects that improve preventive healthcare and lifestyle management. The robust association between innovation and longevity suggests that technological progress directly contributes to healthier, longer lives, confirming the hypothesis that innovation-driven economies are better equipped to achieve sustainable public health gains. This relationship also highlights the role of innovation ecosystems—universities, private research institutions, and health agencies—in translating scientific discovery into tangible health benefits for citizens.

Finally, the relationship between GII and OBCh is statistically insignificant, suggesting that innovation does not exert a meaningful direct impact on behavioral or lifestyle-related health outcomes. Unlike expenditure and longevity indicators, obesity prevalence is primarily influenced by social, cultural, and behavioral factors such as diet, education, and urban lifestyle, which fall outside the immediate scope of technological innovation. This non-significant relationship highlights an important policy gap: while innovation enhances system-level efficiency and clinical outcomes, it may not automatically translate into improved population behaviors or preventive health measures. The result implies that addressing childhood obesity requires complementary strategies—public education, nutrition regulation, and community-based interventions—alongside technological innovation. In other words, innovation alone is not sufficient to improve health behavior; it must be integrated with inclusive and preventive policy frameworks that address the social determinants of health.

**Figure 4:**
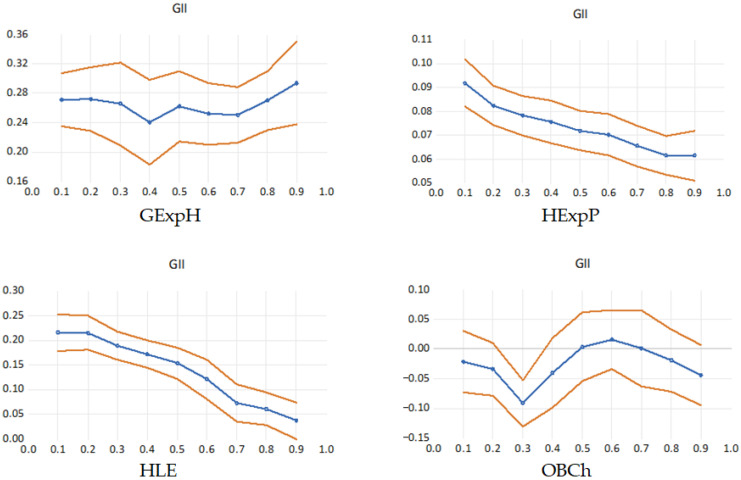
Quantile process of the GII effects on healthcare indicators.

The quantile process presented in Table 6 and graphs in Figure 4 illustrate how the marginal effect of GII varies across different levels of healthcare system performance, providing a more nuanced understanding of the innovation–health relationship beyond the mean estimates. The patterns observed confirm the presence of heterogeneous and nonlinear effects, indicating that innovation does not impact all countries equally but rather exerts differentiated influences depending on their position along the conditional distribution of health outcomes. In this quantile regression process plot, the blue line represents the estimated coefficient of the GII for each quantile of the dependent variable, showing how the impact of innovation varies across the distribution. The orange lines indicate the confidence bands (typically 95%), capturing the uncertainty around these estimates. When the blue line moves outside the orange bands or shows strong variation across quantiles, it signals heterogeneous effects that cannot be detected with standard mean-based regression.

For GExpH, the quantile plot displays a U-shaped pattern, where the positive effect of innovation is stronger at both lower and upper quantiles, but slightly weaker around the median. This implies that innovation stimulates health spending most strongly in countries with either limited or advanced fiscal commitment to healthcare—possibly because innovation acts as a catalyst for reform in less developed systems and as a reinforcement mechanism in already mature systems. In contrast, mid-performing countries may experience a slower fiscal response to innovation due to institutional inertia or transitional policy priorities.

The relationship between GII and healthcare expenditure by provider (HExpP) shows a declining trend across quantiles, suggesting that innovation has a larger marginal effect among countries with lower expenditure levels. This indicates that innovation enhances efficiency and stimulates provider-level resource allocation particularly in underdeveloped or resource-constrained systems, whereas in high-spending health systems, the marginal gains from innovation become less pronounced due to saturation effects or diminishing returns to technology adoption.

For healthy life expectancy (HLE), the quantile coefficients decrease steadily across the distribution, demonstrating that innovation’s contribution to longevity is most significant among lower-performing countries. This downward slope suggests that innovation plays a corrective role in improving health outcomes in states that initially lag behind, supporting the hypothesis of innovation-driven convergence in population health across the European Union. Countries at higher quantiles—where life expectancy is already elevated—experience smaller incremental gains, likely because additional improvements require broader social and behavioral interventions beyond technological advancement.

The relationship with childhood obesity is weaker and statistically insignificant in most quantiles, with coefficients oscillating around zero and only reaching significance at Q0.30 (–0.0912, *p* < 0.001). These findings imply that innovation capacity alone is not a consistent predictor of childhood obesity rates and that other social, behavioral, or nutritional determinants likely play a more dominant role. This behavior reinforces earlier findings that innovation exerts limited direct influence on lifestyle-related health outcomes, such as childhood obesity, which are more strongly shaped by cultural, educational, and socioeconomic factors. The slight improvement in higher quantiles may suggest that advanced innovation systems indirectly contribute to public health awareness or better preventive frameworks, but the overall pattern remains weak and inconsistent.

As an additional robustness check, we estimated a mean fixed-effects OLS model using the same specification. The results reported in Table 7 indicate that innovation capacity remains positively associated with public health expenditure and life expectancy, while reducing childhood obesity prevalence on average. These findings confirm that the observed distributional effects are not artifacts of the quantile framework. However, OLS coefficients mask substantial heterogeneity across the outcome distribution, reinforcing the relevance of quantile modeling for capturing nonlinear and asymmetric relationships across EU health systems.

Beyond statistical significance, the magnitude of the estimated coefficients provides important insights into how innovation translates into measurable health system improvements. For lower-performing countries (e.g., Q10–Q30), the results suggest that a one-point increase in the Global Innovation Index is associated with substantially higher gains in public health expenditure per capita and healthy life expectancy compared to countries at higher quantiles. For instance, GII coefficients for HLE decline from 0.215 at the 10th percentile to 0.038 at the 90th percentile, implying that the same incremental improvement in innovation capacity yields nearly a six-fold larger effect on expected years of healthy life in systems historically constrained by lower investment levels, weaker institutional capacity, and limited technological adoption. These findings highlight that innovation acts as a transformative rather than incremental factor in less advanced systems, accelerating convergence toward higher health performance.

Conversely, the marginal effects observed in higher-performing countries appear significantly smaller, especially for health outcomes. This pattern suggests a saturation effect: once core technological, digital, and institutional infrastructures are in place, further innovation may improve efficiency and specialization rather than producing large population-level health gains. For these systems, innovation contributes more to qualitative improvements—such as digital integration, precision medicine, or improved care coordination—rather than large structural improvements in life expectancy or expenditure allocation. This aligns with the notion that returns to innovation depend on absorptive capacity and the diminishing marginal utility of investments once a performance threshold is reached.

From a policy perspective, these findings imply that innovation-driven health reforms should not follow a uniform blueprint. Countries with low baseline performance may prioritize foundational innovation investments—digital health infrastructure, biomedical R&D capability, and institutional modernization—to unlock rapid gains in public health. In contrast, advanced systems may require targeted innovation strategies focused on cost containment, system integration, or high-complexity medical innovation. Therefore, the magnitude and distribution of effects underscore the need for differentiated policy design rather than treating innovation as a universally linear determinant of health system outcomes.

## 5. Discussion and Policy Implications

The empirical results reveal a differentiated relationship between national innovation capacity and health system performance across EU Member States, demonstrating that innovation does not exert a uniform effect on expenditure, system outcomes, or population health. Instead, the magnitude and direction of these effects vary substantially across quantiles, reflecting heterogeneity in institutional maturity, absorptive capacity, and system readiness. This interpretation aligns with contemporary literature highlighting ecosystem coordination, procurement mechanisms, and governance structures as determinants of innovation outcomes [16,57,58].

The positive and statistically significant association between innovation capacity and public health expenditure across all quantiles suggests that innovation acts as an investment catalyst rather than a cost-containment instrument. Higher coefficients observed at upper quantiles indicate that countries with higher spending levels allocate additional resources toward innovation-driven reforms, consistent with studies on medicines optimization, advanced therapies, and systemic transformation [59,60,61]. At lower quantiles, innovation appears to facilitate foundational capacity building, infrastructure development, and governance strengthening, reflecting early-stage ecosystem formation highlighted in recent evidence from less mature European systems [57,58].

The declining coefficient magnitude for healthcare provider expenditure per capita (HExpP) across higher quantiles indicates that innovation exerts proportionally larger expenditure effects in lower-spending systems while diminishing as systems mature. This matches empirical findings on telemedicine deployment, digital workflow restructuring, and healthcare 4.0 integration, where early adoption incurs higher structural adjustment costs while later stages shift toward efficiency, value optimization, and sustainability [62,63,64,65]. Additionally, studies on digital scaling and integrated care networks support the notion that expenditure growth is front-loaded during initial diffusion phases, whereas advanced systems emphasize optimization rather than expansion [63,64,65].

Healthy life expectancy (HLE) shows a strong positive association with innovation capacity at lower quantiles, tapering off among higher-performing systems. This suggests innovation contributes more prominently to improving population health where baseline clinical performance and access gaps are more pronounced, consistent with research on health outcome convergence in Central and Eastern Europe [66,67]. In contrast, high-performing systems leverage innovation to enhance specialization, precision care, and clinical quality, aligning with evidence from ATMP ecosystems, oncology decision support tools, and neurosurgical AI integration [68,69,70].

The weak and mostly insignificant relationship between innovation capacity and childhood obesity indicates that behavioral health outcomes are not substantially driven by technology-driven system innovation. This corresponds with findings that social determinants, cultural norms, and community-level interventions are more influential in addressing obesity than systemic innovation maturity [67,71]. However, the isolated significant effect at mid-quantiles suggests indirect mechanisms through digital health coaching, VR-based training, and targeted prevention programs, particularly in transitional systems, consistent with emerging evidence on digital public health tools [72,73].

Taken together, the quantile-specific patterns support a multi-stage model of innovation impact. In early stages, innovation drives system-building, capacity expansion, and institutional alignment through procurement platforms, cross-border partnerships, and ecosystem orchestration [16,57,58]. Intermediate stages foster scaling-out, workforce adaptation, and integrated digital service deployment [62,63,64]. Advanced systems focus on specialization, sustainability, and precision, generating incremental value rather than structural change [68,69,70,74]. These dynamics align with conceptual frameworks describing healthcare 4.0 maturity and macro-structural constraints in advanced innovation ecosystems [64,74].

From a policy standpoint, the findings challenge the assumption of uniform innovation returns across the EU. Lower-performing systems exhibit disproportionately large gains, suggesting that policy priorities should center on foundational infrastructure, institutional capacity, and equitable innovation diffusion [58,67,74]. Conversely, high-performing systems may benefit more from policy measures focused on interoperability standards, innovation pricing reform, equitable access to transformative therapies, and sustainability frameworks [61,69,71].

Finally, heterogeneous effects across quantiles underscore the importance of ethical governance, stakeholder coordination, and social alignment. Evidence suggests innovation performance depends not only on technological maturity but also on collaborative governance, ethical oversight, and multi-actor engagement across research, clinical practice, and regulatory domains [57,58,71,74]. Where governance capacity is low, innovation yields structural transformation; where governance is strong, innovation refines performance and specialization.

A supplementary perspective relates to the role of digital and AI-driven technologies in reinforcing public health system resilience. Although the present study examines innovation at a macro-institutional level through GII rather than health-specific technological adoption, recent evidence highlights how digital innovation ecosystems enhance governance capacity, institutional responsiveness, and the management of public health risks through improved information flows and data-driven decision-making. Cianciulli et al. show that artificial intelligence and digital tools can counteract misinformation, strengthen crisis response mechanisms, and support long-term system adaptability, particularly in contexts of public health shocks and digital transformation agendas [75]. Integrating such sector-specific innovation metrics in future research may provide more granular insights into how technological uptake interacts with systemic innovation capacity to influence population-level outcomes.

## 6. Conclusions

This study examined the association between national innovation capacity and healthcare system performance across the European Union using a panel quantile regression approach for the period 2011–2024. The findings suggest that innovation capacity, as measured by the Global Innovation Index, is positively associated with public health expenditure and provider-level spending, although the magnitude and relevance of these relationships vary across the distribution of Member States. Higher effects are observed among countries with lower baseline expenditure, where innovation is more likely to support system modernization and resource expansion, while more developed systems exhibit smaller marginal effects, reflecting efficiency-driven rather than transformative changes.

Healthy life expectancy demonstrates a moderate and heterogeneous association with innovation capacity, particularly in countries where baseline health outcomes are lower. In contrast, minimal statistical association is identified between innovation capacity and childhood obesity, suggesting that behavioral outcomes are driven predominantly by socioeconomic and cultural factors rather than systemic technological capacity. These results indicate that innovation influences structural and institutional dimensions of healthcare performance more strongly than population-level behavioral indicators.

Given the design of the study and the use of aggregate macro-panel data, the results should be interpreted as correlational rather than causal. Although fixed effects and diagnostic testing help mitigate simultaneity and cross-sectional dependence, potential endogeneity and omitted structural variables cannot be fully excluded. It is also important to note that the Global Innovation Index captures broad national innovation capacity rather than health-specific technological advancement, while childhood obesity reflects predominantly behavioral and socio-cultural determinants. As such, the relationships documented in this study should be interpreted with these conceptual boundaries in mind, particularly when comparing systemic performance indicators with lifestyle-driven health outcomes.

Future research may extend this analysis by incorporating sector-specific innovation measures, causal identification strategies, and micro-level data on organizational adoption. Comparative studies that include additional high- and middle-income regions could also contribute to understanding institutional pathways through which innovation shapes healthcare performance under diverse governance models.

While the empirical evidence provides relevant insights into the distributional effects of innovation on healthcare performance in the EU, several limitations must be acknowledged to contextualize the findings. First, the analysis relies on macro-level country indicators, which may mask intra-national inequalities in access, technological adoption, or innovation diffusion, raising the risk of ecological inference limitations. The relationships identified should therefore not be interpreted as describing individual-level or institutional-level behavioral mechanisms. Second, although fixed effects and lagged GII values help mitigate simultaneity concerns, potential endogeneity between innovation capacity and healthcare expenditure cannot be fully excluded, particularly given the bidirectional nature of innovation financing, regulatory reform, and health system modernization. As such, causality should be interpreted cautiously, and future research may benefit from instrumental-variable approaches or structural causal models.

Third, the use of aggregate national innovation performance captures broad technological and institutional capabilities rather than domain-specific advances in health-related innovation. This may underestimate the role of targeted medical research, digital health platforms, pharmaceutical innovation ecosystems, or translational clinical technologies. Consequently, GII should be viewed as a systemic proxy rather than a direct measure of healthcare innovation. Finally, while data were harmonized across sources, differences in coverage across years required restricting observations to the longest common period, which may limit temporal granularity. Future analyses could incorporate sectoral innovation metrics, micro-level datasets, and analytical techniques capable of modeling dynamic feedback loops between innovation, health outcomes, and policy interventions.

## Figures and Tables

**Table 1:** Dataset description.

Indicator	Acronym	Type	Definition	Unit	Years	Source
**General government expenditure on domestic health**	GExpH	Dependent	Public expenditure on health as % of GDP	Percent (%)	2011–2023	Eurostat (SHA 2011 framework) [39]
**Healthcare expenditure by provider**	HExpP	Dependent	Expenditure by healthcare providers per capita	USD PPP/capita	2011–2023	WHO Global Health Expenditure Database [40]
**Healthy life expectancy (at birth)**	HLE	Dependent	Expected years of healthy life from birth	Years	2011–2021	WHO Life Tables [41]
**Obesity in children (5–19)**	OBCh	Dependent	Share of obese children aged 5–19	Percent (%)	2011–2022	World Obesity Atlas/WHO [42]
**Global Innovation Index**	GII	Explanatory	Composite global innovation score	Index (0–100)	2011–2024	WIPO—Global Innovation Index [43]
**GDP per capita growth**	GDP	Control variable	Annual percentage growth of GDP per capita	Percent (%)	2011–2024	World Bank [44]
**Population**	POP	Control variable	National population	Number of inhabitants	2011–2024	World Bank [45]

**Table 2:** Descriptive statistics of the data.

	GEXPH	HEXPP	HLE	OBCH	GII	GDP	POP
Mean	13.5161	2570.949	68.7594	7.4526	48.8863	2.0454	16,704,390
Median	13.3345	2041.870	69.4983	7.5059	48.4900	1.8907	8,916,864.
Maximum	20.9229	6399.520	71.6892	13.2978	64.8000	23.4436	83,196,078
Minimum	5.9832	307.6900	62.3889	3.6975	34.1800	−11.3931	433,481.0
Std. Dev.	3.0368	1674.541	2.1062	2.3466	7.3994	3.9498	21,831,767
Skewness	0.1420	0.3977	−0.7363	0.2116	0.2088	0.2595	1.7984
Kurtosis	2.6935	1.7057	2.3740	2.2138	2.0397	6.7951	4.9989
Jarque–Bera	2.0585	27.2152	30.1908	9.4017	12.9308	173.0118	199.6632
Probability	0.3572	0.0000	0.0000	0.0090	0.0015	0.0000	0.0000
Sum	3825.060	727,578.7	19,458.93	2109.106	13,834.83	578.8527	4.73 × 10^9^
Sum Sq. Dev.	2600.758	7.91 × 10^8^	1250.984	1552.843	15,439.82	4399.628	1.34 × 10^17^

**Table 3:** Residual cross-section dependence test.

Null Hypothesis: No Cross-Section Dependence (Correlation) in Residuals Total Panel (Unbalanced) Observations: 323								
	GExpH		HExpP		HLE		OBCh	
Test	Statistic	Prob.	Statistic	Prob.	Statistic	Prob.	Statistic	Prob.
Breusch-Pagan LM	1582.769	0.0000	2816.416	0.0000	935.392	0.0000	2283.448	0.0000
Pesaran scaled LM	46.490	0.0000	93.051	0.0000	22.05648	0.0000	72.935	0.0000
Pesaran CD	31.336	0.0000	51.708	0.0000	22.11867	0.0000	21.495	0.0000

Note: non-zero cross-section means detected in data. Test employs centered correlations computed from pairwise samples.

**Table 4:** Panel cross-section heteroskedasticity LR test.

Dependent Variable:	GExpH			HExpP			HLE			OBCh		
Test	Value	d.f.	Prob.	Statistic	d.f.	Prob.	Statistic	d.f.	Prob.	Statistic	d.f.	Prob.
Likelihood ratio	168.212	27	0.0000	195.993	27	0.0000	241.579	27	0.0000	327.285	27	0.0000
LR test summary:												
Restricted LogL	−714.671	319		−218.519	329		−570.716	292		−728.332	319	
Unrestricted LogL	−630.565	319		−120.522	329		−449.926	292		−564.689	319	

Note: Null hypothesis: Residuals are homoscedastic.

**Table 5:** Panel quantile regression results.

Dependent Variable:	GExpH		HExpP		HLE		OBCh	
Variable	Coeff.	Prob.	Coeff.	Prob.	Coeff.	Prob.	Coeff.	Prob.
GII	0.2619	0.0000	0.0718	0.0000	0.1534	0.0000	0.0042	0.8858
GDP	0.0853	0.0879	−0.0198	0.0660	−0.1059	0.0406	−0.0718	0.2546
lnPOP	0.3770	0.0251	0.0435	0.0546	−0.0716	0.6077	−0.3210	0.2407
C	−5.2068	0.0758	3.5312	0.0000	62.5429	0.0000	12.4440	0.0125
Pseudo R-squared	0.2696		0.4394		0.2066		0.0064	
Adjusted R-squared	0.2627		0.4342		0.1984		−0.0029	

Note: Estimates correspond to the median quantile (Q50) of the panel quantile regression model. Standard errors are robust to heteroscedasticity and clustered at the country level.

**Table 6:** Panel quantile regression estimates across the conditional distribution.

Quantile	GExpH		HExpP		HLE		OBCh	
	Coefficient	Prob.	Coefficient	Prob.	Coefficient	Prob.	Coefficient	Prob.
**0.10**	0.2714	0.0000	0.0918	0.0000	0.2153	0.0000	−0.0210	0.4225
**0.20**	0.2720	0.0000	0.0823	0.0000	0.2146	0.0000	−0.0337	0.1395
**0.30**	0.2655	0.0000	0.0782	0.0000	0.1889	0.0000	−0.0912	0.0000
**0.40**	0.2406	0.0000	0.0755	0.0000	0.1721	0.0000	−0.0404	0.1792
**0.50**	0.2619	0.0000	0.0718	0.0000	0.1534	0.0000	0.0042	0.8858
**0.60**	0.2519	0.0000	0.0700	0.0000	0.1214	0.0000	0.0161	0.5283
**0.70**	0.2506	0.0000	0.0654	0.0000	0.0731	0.0002	0.0011	0.9732
**0.80**	0.2701	0.0000	0.0615	0.0000	0.0615	0.0003	−0.0192	0.4738
**0.90**	0.2940	0.0000	0.0614	0.0000	0.0380	0.0466	−0.0437	0.0900

**Table 7:** Fixed-effects OLS robustness estimates.

Dependent Variable:	GExpH		HExpP		HLE		OBCh	
Variable	Coeff.	Prob.	Coeff.	Prob.	Coeff.	Prob.	Coeff.	Prob.
GII	0.1412	0.0000	0.0534	0.0000	0.0168	0.0189	−0.1002	0.0000
GDP	0.0557	0.0026	0.0019	0.5144	0.0209	0.0095	−0.0019	0.8594
lnPOP	9.8330	0.0000	−0.0289	0.9336	2.4471	0.0179	0.5842	0.6504
C	−135.3276	0.0001	10.6908	0.0552	29.1427	0.0777	3.1096	0.8801

## Data Availability

https://ec.europa.eu/eurostat/statistics-explained/index.php?oldid=283258&title=Government_expenditure_on_health&utm_source=chatgpt.com (accessed on 7 August 2025); https://apps.who.int/nha/database (accessed on 7 August 2025); https://www.who.int/data/gho (accessed on 7 August 2025); https://www.who.int/health-topics/obesity (accessed on 7 August 2025); https://www.wipo.int/en/web/global-innovation-index (accessed on 2 August 2025); https://data.worldbank.org/indicator/NY.GDP.PCAP.KD.ZG (accessed on 10 August 2025); https://data.worldbank.org/indicator/SP.POP.TOTL (accessed on 10 August 2025).
